# Rhabdomyolysis induced by nelarabine

**DOI:** 10.1007/s00277-022-04879-8

**Published:** 2022-06-17

**Authors:** Christian Späth, Mandy Schönau, Sophie Gaubert, Thomas Neumann, Christian A. Schmidt, Florian H. Heidel, William H. Krüger

**Affiliations:** Clinic for Internal Medicine C – Haematology and Oncology, Stem Cell Transplantation and Palliative Care, University Medicine Greifswald, Ferdinand-Sauerbruch-Straße, 17475 Greifswald, Germany

Dear Editor,

A 56-year-old male suffering from stage IV_B_ (Ann Arbor) T-lymphoblastic lymphoma, karyotype 47, XYY, was induced according to the GMALL elderly protocol with idarubicin, vincristine and dexamethasone [1]. Analysis of CSF revealed no lymphoma cells and intrathecal chemoprophylaxis was performed according to the protocol. Initially, flow cytometry of the bone marrow cells detected 5.5% blasts. Consolidation I (methotrexate and PEG-asparaginase) and consolidation II (high-dose Ara-C) were administered and well tolerated. Computed tomography showed a complete remission after consolidation I and marrow cytology and histology were negative for T-LBL blasts. However, minimal residual disease was still detectable by flow cytometry and by PCR analysis [2, 3].

**Fig. 1:**
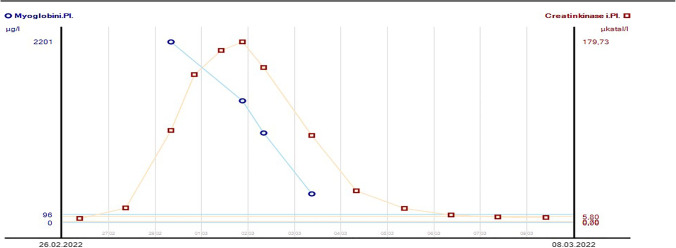
Course of creatinine kinase (orange) and myoglobin (blue) in plasma

Due to high-risk situation, the patient was scheduled for allogeneic stem cell transplantation from a matched unrelated donor and nelarabine was given for bridging. At day 3 of the protocol, the patient reported significant pain in the pelvic/renal region but no signs of renal or coronary disease. However, due to the patient’s BMI of 40.81 the diagnostic focused on exclusion of cardiac disease, but electrocardiography and troponin were negative. Nelarabine was discontinued after two doses. Creatinine kinase (CK) raised to a maximum of 179.73 µkatal/l (upper normal value [UNV] = 5.8 µkatal/l) and myoglobin (MG) to a maximum of 2201 µg/l (UNV = 96 µg/l) (Fig. 1). Lactate dehydrogenase was slightly elevated. The patient’s symptoms disappeared after discontinuation of nelarabine and CK, MG and LDH normalised over the next week under forced diuresis and supportive therapy. Kidney parameters were normal during the entire course. The patient proceeded to allogeneic transplantation in complete remission without further bridging, has been discharged and is well and alive on day + 56 after SCT.

Nelarabine is a nucleoside analogue used for therapy of T-ALL and T-LBL [4, 5]. The main toxicities reported are neurological and haematological. Rhabdomyolysis is a critical illness associated with severe morbidity [6]. The case of rhabdomyolysis as an adverse effect of nelarabine described here is the third in the literature [4, 7, 8]. An unclear increase of creatinine kinase after nelarabine was already reported by Gökbuget et al. in 2011 [4]. In the presented case, the clinical picture in conjunction with the high BMI was first misleading to a suspected cardiac injury. The negative troponin and the positive myoglobin in clinical chemistry delivered finally the diagnosis of a rhabdomyolysis. The early discontinuation of nelarabine was presumably helpful to avoid organ damages and further morbidity.

In conclusion, we suggest a monitoring of myoglobin under nelarabine therapy for early recognition of rhabdomyolysis. A tool for the identification of patients at high risk for rhabdomyolysis would be very helpful.
